# Predictors of Serological Response to SARS-CoV-2 Vaccination in Kidney Transplant Patients: Baseline Characteristics, Immunosuppression, and the Role of IMPDH Monitoring

**DOI:** 10.3390/jcm11061697

**Published:** 2022-03-18

**Authors:** Lutz Liefeldt, Petra Glander, Jens Klotsche, Henriette Straub-Hohenbleicher, Klemens Budde, Bettina Eberspächer, Frank Friedersdorff, Fabian Halleck, Pia Hambach, Jörg Hofmann, Nadine Koch, Danilo Schmidt, Eva Schrezenmeier, Evelyn Seelow, Ulrike Weber, Bianca Zukunft, Kai-Uwe Eckardt, Mira Choi, Friederike Bachmann, Johannes Waiser

**Affiliations:** 1 Department of Nephrology and Medical Intensive Care, Charité—Universitätsmedizin Berlin, Corporate Member of Freie Universität Berlin and Humboldt-Universität zu Berlin, 10117 Berlin, Germany; petra.glander@charite.de (P.G.); henriette.straub@charite.de (H.S.-H.); klemens.budde@charite.de (K.B.); fabian.halleck@charite.de (F.H.); pia.hambach@charite.de (P.H.); nadine.koch@charite.de (N.K.); eva-vanessa.schrezenmeier@charite.de (E.S.); evelyn.seelow@charite.de (E.S.); ulrike.weber@charite.de (U.W.); bianca.zukunft@charite.de (B.Z.); kai-uwe.eckardt@charite.de (K.-U.E.); mira.choi@charite.de (M.C.); friederike.bachmann@charite.de (F.B.); johannes.waiser@charite.de (J.W.); 2 German Rheumatism Research Center Berlin—A Leibniz Institute, 10117 Berlin, Germany; jens.klotsche@drfz.de; 3 Labor Berlin—Charité Vivantes GmbH, 13353 Berlin, Germany; bettina.eberspaecher@laborberlin.com (B.E.); joerg.hofmann@laborberlin.com (J.H.); 4 Department of Urology, Charité—Universitätsmedizin Berlin, Corporate Member of Freie Universität Berlin and Humboldt-Universität zu Berlin, 10117 Berlin, Germany; frank.friedersdorff@charite.de; 5 Business Unit IT, Charité—Universitätsmedizin Berlin, 10117 Berlin, Germany; danilo.schmidt@charite.de; 6 Berlin Institute of Health (BIH), 10178 Berlin, Germany

**Keywords:** kidney transplantation, immunosuppression, SARS-CoV-2 vaccination, serological response, IMPDH monitoring

## Abstract

Immunosuppression increases the risk of severe coronavirus disease 2019 (COVID-19). Morbidity and mortality of this disease in kidney transplant patients are higher than in the general population. As the vaccination response of transplant patients is weak, serological monitoring was performed. In this cohort study, we analyzed the determinants of vaccination response. All patients had no history of COVID-19. With anti-spike IgG monitoring, 148 responders and 415 non-responders were identified. We compared both groups using multivariate analyses of the cohort and a sub-cohort of mycophenolic-acid-treated patients. We investigated the influence of patient characteristics, immunosuppression, and erythrocyte inosine monophosphate dehydrogenase (IMPDH) activity. In responders, the time after transplantation was longer (13.5 vs. 8.5 years), the glomerular filtration rate was higher (56.9 vs. 47.8 mL/min/1.73 m^2^), and responders were younger (53.0 vs. 57.4 years). Heterologous vaccination was more effective than homologous vaccination. Calcineurin inhibitors plus mycophenolate reduced the seroconversion rate. No seroconversion was observed in belatacept patients. In mycophenolate-treated patients, IMPDH activity was a significantly better predictor of response than mycophenolate dose (AUC 0.84 vs. 0.62, *p* < 0.001). Immunosuppression strongly affects vaccine response. Modifications to immunosuppression should be considered in order to facilitate this response. Erythrocyte IMPDH activity can be used to guide mycophenolate treatment.

## 1. Introduction

Recipients of solid organ transplants are at an increased risk of severe disease and death after severe acute respiratory syndrome coronavirus 2 (SARS-CoV-2) infection [1,2]. The responsiveness of kidney transplant patients (KTPs) to SARS-CoV-2 vaccination is severely limited by maintenance immunosuppression [3,4,5,6]. The response rate in heart and liver transplant recipients appears to be higher [7]. Consequently, given the low response rates of KTPs after two vaccine applications, three [8,9,10,11] or more [12] vaccinations may be necessary to achieve a positive serological response. Most KTPs receive triple maintenance immunosuppression consisting of tacrolimus or cyclosporine, mycophenolic acid (MPA), and low-dose steroids. Previous studies have demonstrated that treatment with MPA is a negative predictor of vaccination success [3,6,13], which is also observed in autoimmune diseases, such as systemic lupus erythematosus [14].

MPA dosing usually follows a standard regimen adapted by individual clinical events, such as rejection on one side and gastrointestinal symptoms, hematopoietic side effects, and opportunistic infections on the other side. Because of the well-known dose-response variability of MPA, therapeutic drug monitoring (TDM) has been established, either as MPA blood level or inosine 5′-monophosphate dehydrogenase (IMPDH) monitoring [15,16]. We recently described that IMPDH activity measurement in erythrocytes is a useful strategy for longitudinal monitoring of MPA treatment [17]. The robustness and reproducibility of this method led to routine IMPDH monitoring of all MPA-treated patients in our clinic. IMPDH monitoring has displaced MPA trough level monitoring, as well as the measurement of IMPDH in mononuclear cells. Given that an optimal understanding of vaccination efficacy is essential to protect KTPs against severe coronavirus disease 2019 (COVID-19), we investigated the response rate to SARS-CoV-2 vaccination in a single-center cohort of renal allograft recipients to identify the factors associated with vaccination response, including the potential value of IMPDH activity measurement.

## 2. Patients and Methods

### 2.1. Patients

We searched for all adult (≥18 years) kidney allograft recipients with a functioning graft at Charité–Universitätsmedizin Berlin using our electronic patient record system, TBase [18]. All patients with “complete” vaccination according to the protocol of the manufacturer with authorized vaccines were included, i.e., two applications in the case of Comirnaty^®^ (BioNTech Manufacturing GmbH, Mainz, Germany), Spikevax^®^ (Moderna Biotech, Madrid, Spain), and Vaxzevria^®^ (AstraZeneca AB, Södertälje, Sweden); or one injection in the case of COVID-19 Vaccine Janssen^®^ (Janssen-Cilag International NV, Beerse, Belgium). Combinations of different vaccines were allowed. Further inclusion criteria were an anti-spike IgG antibody test ≥28 days after the last vaccination, no clinical history of COVID-19, and a negative anti-nucleocapsid antibody test. The patients underwent negative serological tests before vaccination. Third vaccine applications led to the exclusion of patients. Positive results for anti-spike IgA only were not classified as seroconversion. The deadline for the database retrieval was 31 August 2021.

### 2.2. Anti-SARS-CoV-2 Antibody Tests

We used an anti-SARS-CoV-2 enzyme-linked immunosorbent assay (ELISA) to detect IgG antibodies against the S1 domain of the SARS-CoV-2 spike (S) protein in serum according to the manufacturer’s instructions (Anti-SARS-CoV-2-ELISA (IgG), EI 2606-9601 G, EUROIMMUN Medizinische Labordiagnostika AG, Lübeck, Germany). Processing and measurement were done using the fully automated Immunomat (Institut Virion\Serion GmbH, Würzburg, Germany). The results were determined by comparing the obtained signals of the patient samples with the previously obtained cutoff value of the calibrator. As suggested by the manufacturer, samples with a cutoff index ≥ 1.1 were considered positive.

To exclude patients who had previously acquired COVID-19, we simultaneously measured antibodies against the nucleocapsid (N protein) using an electrochemiluminescence immunoassay (ECLIA, Elecsys Anti-SARS-CoV-2, 09203079190, Roche Diagnostics GmbH, Mannheim, Germany). The results were determined by comparing the obtained signals of the patient samples with the previously obtained cutoff value of the calibrator. As suggested by the manufacturer, samples with a cutoff index ≥ 1.0 were considered positive.

### 2.3. Erythrocyte IMPDH Activity Measurement

Erythrocyte IMPDH activity was measured as previously described [17]. Briefly, lithium heparin blood was drawn immediately before morning drug intake (pre-dose). Blood was stored at room temperature until cell extracts were prepared (within 24 h). Whole blood (250 µL) was transferred to 15 mL polystyrene tubes filled with 2.5 mL phosphate-buffered saline. After centrifugation at 1200× *g* for 10 min at 20 °C, the supernatant was discarded, and the packed cells were washed with another 2.5 mL of phosphate-buffered saline and centrifuged under the same conditions. The sediment was lysed with 3 mL HPLC-water, and 1 mL aliquots were frozen at −20 °C for later use. IMPDH activity in erythrocytes was measured in the supernatant of thawed cell lysates. We used identical incubation conditions and the previously described HPLC method for mononuclear cells [16]. Xanthosine 5′-monophosphate production was normalized to the hemoglobin concentration. The internal quality control samples were included in each analytical run. The interassay variability for the three different activity levels (1713, 831, and 231 pmol XMP/h/mg hemoglobin) was 4.9%, 5.7%, and 11.3%, respectively. The most recent measurements under steady-state conditions before vaccination were used for the analysis.

### 2.4. Statistical Analyses

Descriptive analyses were performed to describe the parameters of interest using absolute and relative frequencies for categorical data and means with standard deviations or medians with ranges for continuously distributed parameters, as appropriate. The vaccination response rate was analyzed using a multilevel mixed-effects logistic regression model to account for the clustering of patients at the three sites of Charité–Universitätsmedizin Berlin. The medical therapies were divided into mutually exclusive groups. Grand mean coding was performed to analyze the association between vaccination response and therapies and to compare a single therapy with the mean vaccination response across all therapies. Reference-level coding was performed for the other categorical predictor variables. Calcineurin inhibitor (CNI) levels under treatment with tacrolimus or cyclosporine A were combined by calculating the percentage of deviation from the base 6 ng/mL for tacrolimus and 80 ng/mL for cyclosporine A.

Receiver operating characteristic (ROC) analysis was used to evaluate the predictive ability of MPA dose, lymphocyte count, and IMPDH activity in relation to response to SARS-CoV-2 vaccination in patients treated with MPA. The area under the ROC curve (AUC) was compared using the statistical test proposed by DeLong et al. [19]. ROC analyses were used to estimate the cutoff for IMPDH using the Youden index. The resulting cutoff value maximizes the sum of the sensitivity and specificity to predict the response to vaccination. All statistical analyses were performed using STATA version 12.1.

## 3. Results

**Figure 1:**
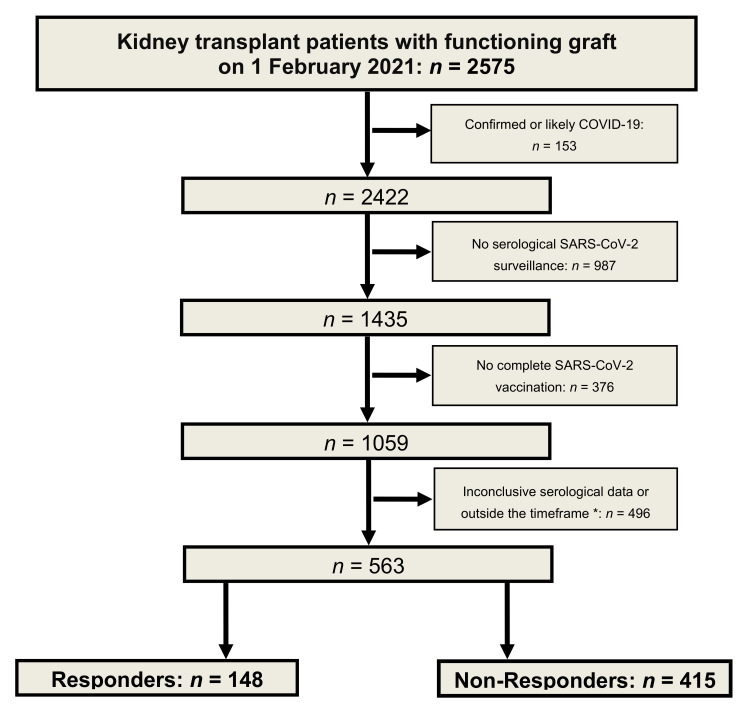
Algorithm for the definition of a cohort of responders and non-responders after standard SARS-CoV-2 vaccination in kidney transplant patients. * Time frame: ≥28 days after the second vaccination but before third vaccination or SARS-CoV-2-infection (end of observation: 31 August 2021).

The algorithm used to establish the cohort is shown in Figure 1. Based on the availability of serological, vaccination, and medication data in our electronic patient record system, we identified 148 responders and 415 non-responders to standard SARS-CoV-2 vaccinations. The baseline characteristics of the study cohort are summarized in Table 1.

The responders were younger and had better graft function. The interval between transplantation and vaccination was longer in responders. The gender distribution was similar between responders and non-responders.

The application of two doses of BioNTech-Pfizer vaccine was dominant in our cohort. A small subset of the cohort was administered a heterologous vaccination (mRNA after vector vaccine). The patients had been administered Vaxzevria^®^, and due to increasing safety concerns for the second application, an mRNA-based vaccine had been chosen. Among the non-responders, the majority received three-fold immunosuppression, whereas responders were similarly often two- and three-fold immunosuppressed.

Tacrolimus in combination with MPA is the most common immunosuppressive regimen. Altogether, 49.5% of patients received immunosuppressive doses of glucocorticoids (≥5 mg prednisolone equivalent daily).

Multivariate analyses were performed to determine the influence of baseline characteristics, vaccine types, immunosuppressive combinations, eGFR, lymphocyte counts, and CNI trough levels on response to SARS-CoV-2 vaccination (Table 2).

Higher age increased the risk of non-response to vaccination, whereas longer time after transplantation significantly increased the serological response rate. The selection of vaccine(s) influenced the success of vaccination. In our cohort, we found the highest immunogenicity with heterologous vaccine combinations. Immunosuppressive combinations containing MPA (tacrolimus + MPA ± steroid, cyclosporine + MPA ± steroid, and belatacept + MPA ± steroid) significantly decreased the odds of serological response after SARS-CoV-2 vaccination. In contrast, the absence of MPA or replacement of MPA with other immunosuppressants increased the probability of response. A higher eGFR, higher lymphocyte count, and lower CNI trough levels were also associated with higher response rates. However, steroids did not have convincing effects on humoral immune reactions after vaccination.

For a detailed analysis of the determinants of vaccination response in patients receiving MPA, we analyzed a sub-cohort of 492 patients with MPA (Table 3).

As before, younger age and a longer interval between transplantation and vaccination increased the probability of response in this subgroup. In addition, male gender had a positive impact on the response rate. The inclusion of MPA dose and IMPDH activity measurements in the analysis led to the loss of significance of the vaccine(s), most of the immunosuppressive combinations (except MPA + Tac + steroid), and lymphocyte counts with respect to the serological response rate. Higher eGFR and lower CNI trough levels moderately improved the humoral response to vaccination. Markers of MPA exposure, i.e., a lower MPA dose and lower erythrocyte IMPDH activity, were associated with significantly increased response rates.

**Figure 2:**
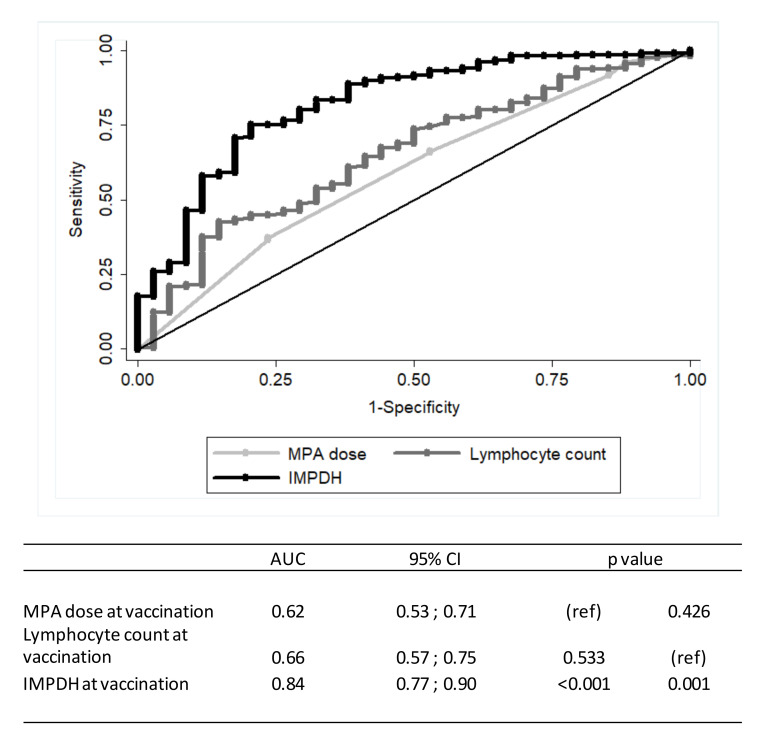
Predictive performance for response to SARS-CoV-2 vaccination after kidney transplantation (*n* = 348 MPA-treated patients with IMPDH measurement). (ref) = reference category.

The correlation between IMPDH activity and MPA dose was weak (r = 0.17, *p* = 0.015). ROC analyses were performed to compare the influence of MPA dose and IMPDH activity on the prediction of response to SARS-CoV-2 vaccination (Figure 2).

## 4. Discussion

The need for better vaccination strategies led us to analyze our kidney transplant patients with respect to their humoral response after standard SARS-CoV-2 vaccination. For this purpose, we identified clearly defined responders and non-responders based on the detection of anti-spike IgG antibodies and sought factors with a significant influence in multivariate analyses.

We focused on serological results in our cohort because of the broad availability of data and the lack of more sophisticated assays, such as neutralizing antibody assays and cellular immunity assays, in the majority of patients. Although the value of serological response after SARS-CoV-2 vaccination was initially questioned, German health authorities have accepted and recommended this monitoring in immunocompromised patients, including following solid organ transplantation [20]. The spectrum of assays used to monitor the immunological response to SARS-CoV-2 vaccination is broad, and recent studies have suggested significant differences between humoral and cellular immunity [21]. The question of protection against SARS-CoV-2 infection in relation to the extent of humoral responses after vaccination was not the focus of our analysis but is an important topic for future studies. Our serological approach is supported by the increasing burden of breakthrough infections [22,23] and data on the kinetics of antibody responses over time (neutralizing antibodies and total antibody concentrations) for the estimation of the duration and degree of protection provided by vaccines [24,25].

As expected, several patient characteristics have an impact on the serological response after vaccination: older patients, more recently grafted patients, and those with poorer kidney graft function are at a higher risk of non-response. Age and lower GFR were also predictive of fatal outcomes in a Brazilian cohort used to establish a tool for the early prediction of COVID-19-associated death after kidney transplantation [26], and immunosenescence likely played a role in both observations [27]. Beyond patient age, the intensity of early immunosuppression with high rates of triple immunosuppression, higher MPA exposure, higher CNI trough levels, and various induction regimens with monoclonal or polyclonal antibodies (data not shown) favored non-response to vaccination. The lower rate of antibody formation after vaccination in patients with lower lymphocyte counts is in accordance with data from a recent French study [8].

Soon after the approval of mRNA- and vector-based SARS-CoV-2 vaccines, the concept of heterologous vaccination with both types of vaccines was discussed based on retrospective data from non-renal patients [28]. Data from a prospective study of healthcare workers at our institution further supports this concept [29]. The significantly higher response rate of KTPs vaccinated with heterologous combinations in our cohort is in accordance with the data from non-renal patients.

Patients receiving immunosuppressive combinations including MPA had significantly lower response rates to SARS-CoV-2 vaccination than those without MPA. Among patients treated with MPA, 18.7% were responders, compared to 78.9% of patients without MPA. The magnitude of the problem was first described by Segev et al. in May 2021 using a social-media-based study of solid organ recipients [13] and subsequently confirmed in other studies [3,6]. The combination of belatacept and MPA in not a single case enabled B lymphocytes to mount a measurable antibody response after vaccination in our cohort. In a French study, even the administration of a third dose of BNT162b2 mRNA COVID-19 vaccine did not improve immunogenicity in KTPs treated with belatacept [30].

The negative effect of proliferation inhibition with MPA on vaccination response led us to perform a more detailed analysis of the sub-cohort of MPA-treated patients. In the multivariate analysis, age, time interval between transplantation and vaccination, and GFR remained significant predictors of response, whereas vaccine combinations and most immunosuppressive regimens lost their influence. The significant disadvantage of female patients probably reflects a higher susceptibility to MPA effects. The MPA dose as a marker of short-term exposure and IMPDH activity as a marker of medium-term MPA exposure clearly dominated the results of the multivariate analysis. In a head-to-head comparison between MPA dose and IMPDH activity, the latter proved to be a significantly better predictor of serological response after complete SARS-CoV-2 vaccination in KTPs. These data suggest that dose modification of MPA is a strategy to improve response rates after vaccination, supporting the conclusions of other studies [31].

Our study had some limitations. This was a retrospective analysis based solely on serological monitoring after only two vaccine applications. The cohort represents a broad spectrum of patient characteristics, vaccines, and immunosuppressive regimens, rendering the analysis a real-world study. In contrast, the classification of patients into two response groups resulted from a strict selection process. The number of patients with respect to lymphocyte counts was low (62.9%), and IMPDH monitoring was incomplete (348 of 492 patients).

After 31 August 2021 (deadline for the database retrieval), several patients were vaccinated a third time (usually without prior reduction in immunosuppression) or even a fourth time (usually with reduced immunosuppression). The former did not significantly improve the response rate and therefore has not been included in this analysis. The results of the latter are still not available (short followup).

In summary, elderly patients with low GFR are at high risk of non-response to standard SARS-CoV-2 vaccination. Strong immunosuppression or overimmunosuppression should be avoided to ensure successful vaccination. MPA and belatacept are highly potent in preventing humoral immune responses to vaccination. Heterologous vaccination strategies elicited the highest response rates in our cohort. Tailoring MPA-based immunosuppression using IMPDH monitoring may be a promising strategy for successful vaccination against SARS-CoV-2 after kidney transplantation. Another strategy might be the complete peri-vaccination cessation of MPA, as already done in non-renal patients [32] and planned in a prospective study with KTPs in Israel [33].

## 5. Conclusions

Our data suggest a risk-based stratification to obtain a sufficient immune response to SARS-CoV-2 vaccination and an effective immunosuppression in KTPs:

First, given the observed lack of response in patients treated with belatacept, the drug should not be initiated unless patients are immunized against SARS-CoV-2.

Second, in patients already treated with belatacept, a temporary switch to another immunosuppressive strategy before the first vaccination attempt is warranted. If this is impossible, a passive vaccination strategy might be advisable.

Third, in patients treated with dual immunosuppression (e.g., CNI plus steroids), an attempt to achieve a vaccination response without prior modification of immunosuppression is warranted.

Fourth, in patients treated with MPA, dose reduction and the best available TDM appear to be important for optimizing the vaccination response. In addition, given the association of dose reductions or discontinuation of mycophenolate with an increased risk of acute rejection and inferior patient and graft survival [34], a precise TDM may increase safety and should be the basis of such maneuvers. To our knowledge, this is the first report to show that erythrocyte IMPDH activity measurements are helpful in predicting vaccination responsiveness in MPA-treated patients. Given the reduced mortality of Omicron and new therapeutic options for COVID-19, one should balance the risks of reduction in immunosuppression for successful vaccination against risks of SARS-CoV-2 infection of vulnerable patients.

## Figures and Tables

**Table 1:** Patient characteristics of responders and non-responders after vaccination against SARS-CoV-2.

Characteristic	Total	Responder	Non-Responder
	(*n* = 563)	(*n* = 148)	(*n* = 415)
**Age at vaccination (years)**			
Median (range)	58.0 (18–88)	52 (18–81)	59 (19–88)
Mean (standard deviation)	56.2 (14.2)	53.0 (13.8)	57.4 (14.2)
**Gender**			
Female (number; %)	226; 40.1	58; 39.2	168; 40.5
**Time after transplantation (years)**			
Median (range)	8.3 (0.3–37.7)	11.5 (0.7–37.7)	7.8 (0.3–31.3)
Mean (standard deviation)	9.8 (7.5)	13.5 (9.0)	8.5 (6.5)
**eGFR at vaccination (mL/min/1.73 m²)**			
Median (range)	50 (9–98)	56 (13–95)	47 (9–98)
Mean (standard deviation)	50.2 (20.2)	56.9 (18.9)	47.8 (20.1)
**Vaccination (number; %)**			
BioNTech-Pfizer	389; 69.1	97; 65.5	292; 70.4
Moderna	60; 10.7	20; 13.5	40; 9.6
AstraZeneca	72; 12.8	12; 8.1	60; 14.4
Johnson & Johnson	1; 0.2	0; 0	1; 0.2
Mixed	41; 7.3	19; 12.8	22; 5.3
**Number of immunosuppressants at vaccination**			
1 (number; %)	5; 0.1	4; 2.7	1; 0.2
2 (number; %)	208; 36.8	72; 48.6	136; 32.8
≥3 (number; %)	350; 62.1	72; 48.6	278; 67.0
**MPA treatment at vaccination (number; %)**	492; 87.4	92; 62.2	400; 96.4
**MPA dose (mg/day) mean (SD)**	1442.6 (495.5)	1.244.6 (518.8)	1.488.1 (479.2)
**Co-immunosuppression at vaccination**			
Tacrolimus (number; %)	395; 70.2	94; 63.5	301; 72.5
Tacrolimus trough level (ng/mL); mean (SD)	6.1 (1.5)	5.7 (1.3)	6.3 (1.6)
Cyclosporine (number; %)	102; 18.1	43; 29.0	59; 14.2
Cyclosporine trough level (ng/mL); mean (SD)	100.8 (32.2)	93.7 (20.6)	106 (37.9)
Belatacept (number; %)	45; 8.0	0; 0	45; 10.8
Other: AZA (number; %)	22; 3.9	17; 11.5	5; 1.2
Other: mTORi (number; %)	17; 3.0	12; 8.1	5; 1.2
**Steroid treatment**			
All (number; %)	398; 70.7	106; 71.6	292; 70.4
Dose ≥5 mg prednisolone equivalent daily (number; %)	279; 49.6	70; 47.3	209; 50.4

**Table 2:** Univariate and multivariate analysis of factors associated with serological response to SARS-CoV-2 vaccination after kidney transplantation.

		Responders		Non-Responders		Responder versus Non-Responder					
		*n* = 148		*n* = 415		Univariate			Multivariate		
		*n*	%Row	*n*	%Row	OR	95% CI	*p* Value	OR	95% CI	*p* Value
Female		58	25.7	168	74.3	0.99	0.67; 1.46	0.965	0.75	0.46; 1.21	0.240
Age at 2nd vaccination ^1^		53.0 (13.8)		57.4 (14.2)		**0.98**	**0.97; 0.99**	**0.002**	**0.98**	**0.96; 1.00**	**0.039**
Time after kidney transplantation (years) ^1^		13.5 (9.0)		8.5 (6.5)		**1.09**	**1.06; 1.12**	**<0.001**	**1.06**	**1.02; 1.10**	**0.001**
Vaccination											
AstraZeneca—AstraZeneca		12	16.7	60	83.3	0.64	0.33; 1.25	0.192	0.63	0.27; 1.47	0.287
Heterologous scheme ^2^		19	47.3	22	53.7	**2.53**	**1.31; 4.90**	**0.006**	**2.99**	**1.31; 6.82**	**0.009**
BioNTec-Pfizer—BioNTec-Pfizer		97	24.9	292	75.1	1.00			1.00		
Moderna—Moderna		20	33.3	40	66.7	1.44	0.80; 2.60	0.227	1.89	0.91; 3.90	0.086
	Other	0	0.0	1	100.0	-	-	-	-	-	-
Tac + MPA + Steroid		20	11.5	154	88.5	**0.15**	**0.09; 0.25**	**<0.001**	**0.15**	**0.08; 0.28**	**<0.001**
Tac + MPA - Steroid		42	23.1	140	76.9	**0.49**	**0.32; 0.75**	**0.001**	**0.38**	**0.23; 0.64**	**<0.001**
CyA + MPA ± Steroid		22	29.0	54	71.1	0.60	0.35; 1.02	0.059	**0.51**	**0.27; 0.96**	**0.038**
Belatacept + MPA ± Steroid		0	0.0	42	100.0	-	-	-	-	-	-
Tac/CyA + Azathioprine—Steroid		16	80.0	4	20.0	**3.46**	**1.43; 8.32**	**0.006**	**4.22**	**1.51; 11.83**	**0.006**
mTOR + MPA ± Steroid		6	54.6	5	45.5	0.80	0.27; 2.35	0.679	0.79	0.24; 2.63	0.706
Tac/CyA + Steroid		25	78.1	7	21.9	**4.07**	**1.95; 8.50**	**<0.001**	**4.11**	**1.71; 9.90**	**0.002**
eGFR at vaccination (mL/min/1.73 m²) ^1^		56.9 (18.9)		47.8 (20.1)		**1.02**	**1.01; 1.03**	**<0.001**	**1.03**	**1.02; 1.04**	**<0.001**
lymphocyte count at vaccination ^1^		1734 (1,360)		1325 (582)		**1.07**	**1.03; 1.12**	**0.001**	**1.12**	**1.06; 1.18**	**<0.001**
CNI trough levels at vaccination in % (deviation) ^1,3^		102.2 (24.8)		109.1 (32.7)		**0.96**	**0.93; 0.99**	**0.034**	**0.94**	**0.90; 1.00**	**0.036**

CI = confidence interval, eGFR = estimated glomerular filtration rate, MPA = mycophenolic acid, OR = odds ratio, CNI = calcineurin inhibitor. ^1^ Odds ratio for increase by one unit, except for lymphocytes (by 100 units) and CNI trough levels (%deviation, by 5%). ^2^ AstraZeneca—BioNTech-Pfizer (*n* = 28), AstraZeneca—Moderna (*n* = 13). ^3^ Deviation in % from base 6 ng/mL for tacrolimus and 80 ng/mL for cyclosporine A trough levels.

**Table 3:** Univariate and multivariate analysis of factors associated with serological response to SARS-CoV-2 vaccination in MPA-treated patients after kidney transplantation.

		Responders		Non-Responders		Responder versus Non-Responder					
		*n* = 92		*n* = 400		Univariate			Multivariate		
		*n*	%Row	*n*	%Row	OR	95% CI	*p* Value	OR	95% CI	*p* Value
Female		28	14.7	162	85.3	0.65	0.40; 1.06	0.085	**0.41**	**0.20; 0.83**	**0.013**
Age at 2nd vaccination		51.7 (12.1)		57.3 (14.2)		**0.97**	**0.96; 0.99**	**0.001**	**0.96**	**0.94; 0.99**	**0.002**
Time after kidney transplantation (years)		12.3 (7.6)		8.3 (6.2)		**1.09**	**1.05; 1.13**	**<0.001**	**1.07**	**1.01; 1.13**	**0.031**
Vaccination											
AstraZeneca—AstraZeneca		5	8.1	57	91.9	0.41	0.16; 1.06	0.066	0.53	0.15; 1.86	0.323
Heterologous scheme ^1^		14	40.0	21	60.0	**3.08**	**1.48; 6.40**	**0.003**	2.86	0.90; 9.05	0.074
BioNTec-Pfizer—BioNTec-Pfizer		61	17.8	282	82.2	1.00			1.00		
Moderna—Moderna		12	23.5	39	76.5	1.42	0.70; 2.88	0.330	2.53	0.90; 7.08	0.077
	Other	0	30.8	1	100.0	-	-	-	-	-	-
Immunosuppression											
MPA + CyA ± Steroid		22	29.0	54	71.1	1.32	0.81; 2.14	0.262	1.30	0.57; 2.98	0.534
MPA + Tacrolimus - Steroid		41	22.7	140	77.4	1.05	0.70; 1.56	0.823	1.11	0.58; 2.15	0.750
MPA + Tacrolimus + Steroid		20	11.5	154	88.5	**0.36**	**0.22; 0.58**	**<0.001**	**0.43**	**0.20; 0.95**	**0.036**
MPA + Belatacept ± Steroid		0	0.0	42	100.0	-	-	-	-	-	-
eGFR at vaccination (mL/min/1.73 m²) ^2^		59.3 (17.4)		48.2 (19.6)		**1.03**	**1.02; 1.04**	**<0.001**	**1.03**	**1.01; 1.05**	**0.014**
Lymphocyte count at vaccination ^2^		1781.1 (1597.6)		1321.4 (582.1)		**1.14**	**1.08; 1.20**	**<0.001**	1.06	0.99; 1.14	0.077
CNI trough levels at vaccination (% deviation) ^2,3^		101.8 (23.8)		108.9 (32.7)		**0.95**	**0.91; 1.00**	**0.038**	**0.92**	**0.84; 1.00**	**0.040**
MPA dose at vaccination (MMF equivalent in g/day) ^2^		1244.6 (518.8)		1488.1 (479.2)		**0.78**	**0.69; 0.88**	**<0.001**	**0.72**	**0.59; 0.87**	**0.001**
IMPDH activity ^2^		595.1 (437.2)		1209.6 (614.3)		**0.29**	**0.22; 0.37**	**<0.001**	**0.34**	**0.25; 0.46**	**<0.001**

CI = confidence interval, eGFR = estimated glomerular filtration rate, IMPDH = inosine monophosphate dehydrogenase, MPA = mycophenolic acid, OR = odds ratio, CNI = calcineurin inhibitor. ^1^ AstraZeneca—BioNTec-Pfizer (*n* = 23), AstraZeneca—Moderna (*n* = 12). ^2^ Odds ratio for increase by one unit, except for lymphocytes (by 100 units), MPA dose (by 250 mg/day), IMPDH (by 300 units), and CNI trough levels (%deviation, by 5%). ^3^ Deviation in % from base 6 ng/mL for tacrolimus and 80 ng/mL for cyclosporine A.

## Data Availability

The data presented in this study are available on request from the corresponding author. The data are not publicly available.
